# Supplementary material for: Decomposing Simon task BOLD activation using a drift-diffusion model framework
Source: Sci Rep. 2020 Mar 3;10:3938. doi: 10.1038/s41598-020-60943-1 (PMC7054266; doi:10.1038/s41598-020-60943-1)
Supplement: Supplementary file 1 — Supplementary Information. [file 41598_2020_60943_MOESM1_ESM.pdf]

Decomposing Simon task BOLD activation using a drift-diffusion model framework

James R. McIntosh, Paul Sajda

Supplementary Material

SE-SSM

Full model

In previous work[1] we defined a model to capture the Simon task which we reproduce here with parameters consistent with the present manuscript:

$$\Delta x = v \cdot H(1 + c \cdot b \cdot d)\Delta t + s\xi_1\sqrt{\Delta t} \quad (1)$$

$$x_0 = x_b \cdot L \quad (2)$$

$$t^{eff} = t + \xi_2 s_t \quad (3)$$

Where the conflict $c = \frac{1-L\cdot H}{2}$, and H and L are trial dependent variables with values of -1 or $+1$ corresponding to the required handedness of the response (color), and visual hemifield of the stimulus respectively. All other parameters are defined as presented in the main text, although we would also like to note that we refer to the parameter b as conflict counteraction as opposed to attention.

The model used in this manuscript removes the explicit dependence on L and H, and is instead written in terms of the presence of conflict.

Model fitting toolbox

In order to fit the SE-SSM, as well as the various nested models and extensions provided, we developed an Matlab toolbox (https://github.com/jrmxn/malleable-ssm). For parameter initialization, this toolbox incorporates some core functions from the HDDM toolbox[2] translated from Python to Matlab, as the speed of these methods[3] is unmatched for the SSM. However, in order to generate density estimates with which to calculate likelihoods for our non-standard SSM candidates, we resorted to encoding the probability distributions of the decision-variable given the value into a transition matrix. The toolbox allows for rapid modification and testing of new models by inheriting from the base SSM class, and then redefining the density estimation.

Transition matrix approach[4]

In order to generate the probability density of the response time distribution for a specific set of parameters and conditions we resorted to encoding the probabilistic model decision-variable updates over time into a transition matrix (a similar method was employed by Brunton et al. 2013[5]). We may at first consider trying to estimate $p(x_t|x_{t-1})$ iteratively in order to calculate $p(x > x_{th};t)$, i.e. the probability of being above the decision threshold at any specific time point (Figure S1a). From the SE-SSM equation, we know that $E(x_t|x_{t-1}) = x_{t-1} + v(1 + c \cdot b \cdot d)\Delta t$ and $Var(x_t|x_{t-1}) = s^2\Delta t$, we can therefore discretize the decision variable, and structure a matrix of the form $A_{ij} = p(x_t^j|x_{t-1}^i)$, which given a starting condition x_0, can be multiplied by itself iteratively:

$$p(x|t) = (A')^q x_0$$

where $q = t/\Delta t$.Thus far, this formulation ignores the threshold crossing, which can be introduced by stopping the decision variable from taking a value below the threshold once it has been above it (and vice versa). We can implement this for the upper bound by forcing $p(x_t > x_{th}|x_{t-1} > x_{th}) = 1$ by modifying the A matrix appropriately, and performing the equivalent procedure for the lower bound (Figure S1b). A single pass of recursive multiplication of the A matrix given initial conditions then generates the cumulative response time distribution for a set of parameters, which can then be numerically differentiated to generate the response time distribution. Example usage is included in the SE-SSM Matlab toolbox.

Model fitting evaluation

In the ideal case where the underlying behavioral data really comes from a process which is captured by our model, we sought to confirm that our parameters could be recovered with sufficient confidence.

In order to do this, we took our previously fitted parameter values and sampled from each subject the number of trials that they performed. To replicate the procedure described in the manuscript, we repeated this fit with random starts five times. We also repeated this procedure three times to gauge the variability induced by the limited number of samples available for each subject.

We found that as shown in Figure 2, the parameters are well recovered, with correlations for the parameters between true and recovered model being generally greater than 0.8. The parameter that is recovered least well is the conflict counteraction

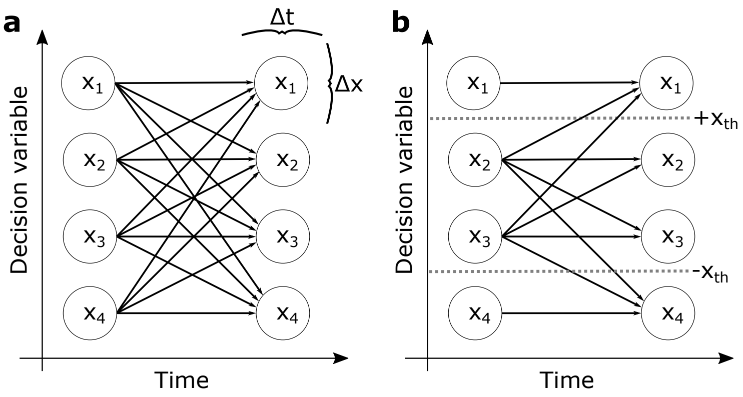

Figure S 1. Depiction of decision variable transitions for response time distribution estimates. (**a**) Example of decision-variable transitions capturing $p(x_t|x_{t-1})$. (**b**) Branches are pruned to incorporate threshold crossings. Note that in the decision variable is discretized at a much finer scale than is depicted here (see main text).

parameter b, as expected since this is effectively operating with half the trials since it is only relevant in the presence of conflict by our model definition.

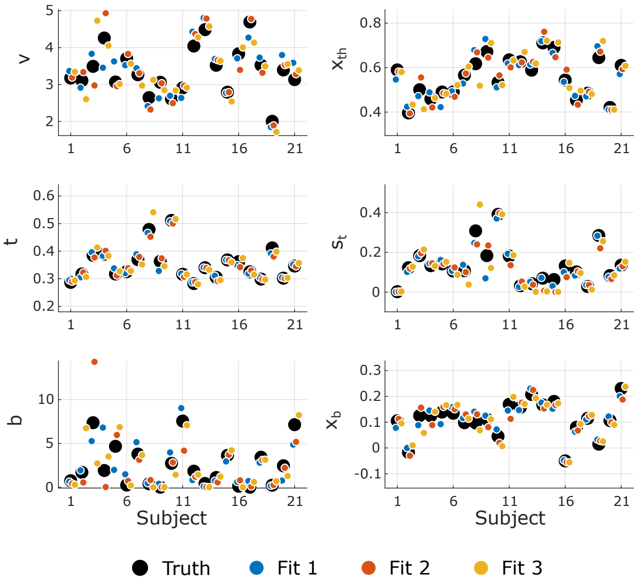

Figure S 2. Model fitting parameter recovery. Black circles mark the original underlying parameter fits. Yellow, orange and blue circles mark recovered parameters, with the colors indicating the different samples. Multiple presentations of identically colored circles indicate repeated fit attempts with different random starts.

Additional analyses

We pursued two additional analyses which we include here for completeness.

Across trial BOLD estimates

We were interested in estimating model parameter fluctuations across trials, which we consider to be likely related to learning processes, stimulus strength, as well as random fluctuations in performance. Regions involved in adaptation across trials (modeled as changes in SE-SSM) should either directly correspond to regions that are activated within trial (for example, they may be changes in recurrent excitation in decision circuits) or project to these regions. Examples of such regions are SMA and lPFC, which may also be involved in conflict counteraction and the specification of control signals in response to conflict. While stimulus strength is fixed throughout the experiment, noise in its cortical representation may be present, and this would conceivably manifest in visual areas.

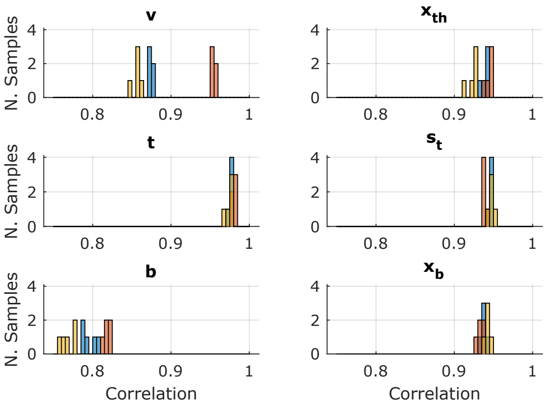

Figure S 3. Model fitting recovery correlation of original parameters with fitted parameters. Each color (see Figure S2) represents a different sample from the original model, with each correlation value stemming from a comparison between the original model parameter and the recovered parameters initiated with a different random start.

The methods we have introduced thus far enable us to look at brain regions that appear related to our parameters across subjects, and to search for activity correlated with the expected decision-variable at different times within a trial. In order to examine the temporal domain between these, we investigated whether average deconvolved activity in individual voxels could be used to augment the SE-SSM on a trial by trial basis. Deconvolution was carried out using the 'least squares - separate' (LS-S) method[6]. For our purposes, this involves repeating GLM fits for each trial, where for each model fit we assign one regressor to a single event matching the stimulus presentation on the trial of interest, and another regressor to absorb all other stimulus events. The regression parameter corresponding to our trial of interest is then treated as the deconvolved activation for this specific trial.

For this analysis, we considered several extensions to the base SE-SSM, for example the augmentation of the drift parameter in a trial dependent manner:

$$\Delta x = (v + g_v r_i)(1 + c \cdot b \cdot d)\Delta t + s\xi_1\sqrt{\Delta t} \tag{4}$$

$$x_0 = x_b(1 - 2c) \tag{5}$$

$$t^{eff} = t + \xi_2 s_t \tag{6}$$

Where in equations 4-6, r_i is a value extracted from trial i of an fMRI derived measure. For example, r_i may be the average activation of deconvolved BOLD activation in a specific voxel for trial i. The parameter g_v acts to scale the individual trial contributions r_i of these fMRI derived measures.

Two computational difficulties arise when attempting to estimate individual trial parameters based on individual trial voxel activation. Firstly, due to the individual trial modulations, the model likelihood is now slightly modified on each trial, and consequently has to be computed for each trial as opposed to each condition. This is problematic as the time to calculate the likelihood for a set of parameters then scales with the number of trials. Secondly, if a whole brain analysis is required, then this model fit has to be performed at each voxel, which is also impractical. To address these two problems, we make the following compromise: we fix the model parameters at their final estimate (as described in the main text), and then introduce the scaling parameter for r_i (i.e. g_v in the example above). We then estimate the probability density function (dependent on RT, conflict, correctness) for different linearly scaled, zero centered propositions of the impact of the scaling parameter (i.e. $g_v r_i$ propositions in the example above). The densities are then made independent of RT, conflict and response by conditioning on the subject response data. Finally, for each voxel we attempt to estimate the parameter g_v by indexing into our candidate density functions with $g_v r_i$ and maximizing the sum of the log-likelihood over trials.

After conducting this analysis for each subject, we can then perform a TFCE permutation analysis, although rather than calculating z-transformed correlations (as in the main text) we calculate the z-statistics directly, and compare to permutations generated by randomizing the sign of the parameter across subjects.

We repeated this analysis for individual trial modulation of the bias parameter x_b, the conflict counteraction parameter b, the model threshold x_{th}, and the drift parameter v.

While we found no clear coupling for bias and threshold, both conflict counteraction and drift result in substantial activation across cortical regions.

Small changes in drift and conflict counteraction both lead to similar changes in RT, and it is therefore not possible to say whether one parameter is playing a role while the other is not. Additionally, conflict counteraction is associated with less data (because its effect is zero on non-conflict trials) so a direct comparison is difficult to interpret. Having said this, we repeated this analysis while optimizing drift and conflict counteraction simultaneously and found that no conflict counteraction clusters remained significant. We take this as an indication that the trial-to-trial fluctuations are dominated by changes in drift. Particular regions of interest that are activated in this analysis are cingulate, cuneal, precuneal and posterior cingulate, as well as Superior temporal gyrus (STG), Inferior frontal gyrus (IFG), Medial frontal gyrus (MFG) and the inferior parietal lobule.

In this analysis, regions involved in adaptation across trials modeled as changes in SE-SSM parameters should either directly correspond to regions that are activated within trial (e.g. they are changes in recurrent excitation in decision circuits) or project to these regions. We were expecting pre-SMA activation as well as DLPFC activation, and potentially dACC. We found that these were indeed activated, although other activation was also present. Other activated regions may simply represent trial-to-trial differences in stimulus processing which are irrelevant to the Simon task. It is also worth noting that on an individual trial, changes in drift and changes in conflict counteraction lead to similar changes in RT, making it hard to attribute specific regions to particular processes.

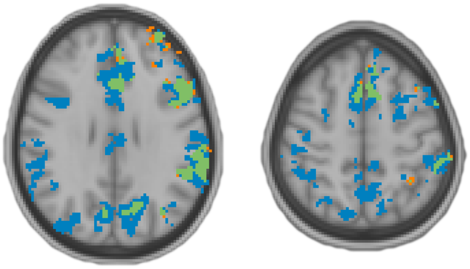

Figure S 4. Across trial parameter fluctuation analysis. Blue: positive activation ($p < 0.05$, GFP FWER corrected) related to the drift term, orange: related to the conflict counteraction parameter, green: related to both conflict counteraction and drift parameters. No negative activation was found with this analysis.

History model

It is known that presence of conflict influences RT in subsequent trials[7,8]. We therefore extended the SE-SSM with history terms:

$$\Delta x = (v + v_h r_{i-1})(1 + c \cdot (b + b_h r_{i-1}) \cdot d)\Delta t + s\xi_1 \sqrt{\Delta t} \tag{7}$$

$$x_0 = (x_b + z_h a r_{i-1})(1 - 2c) \tag{8}$$

$$t^{eff} = t + \xi_2 s_t \tag{9}$$

Where $r_i = c_i - 0.5$ represents the presence of conflict on the previous trial, and v_h, b_h and z_h represent parameters to be fitted. We initially fitted each candidate history parameter independent of the other, however to exclude the possibility that both z_h and b_h are needed for the fit, we additionally ran this combination together.

We found that including history into our model, indeed improved the overall model fit quality as shown in 5a. In particular, the model including z_h was favored over including b_h and v_h as well as over the SE-SSM. We wondered whether z_h and b_h together in the same model might improve the fit quality further, but found this not to be the case.

Given that the fitted z_h are on average negative for our subjects, the interpretation is that when a previous trial was a conflict trial, a mechanism to reduce the bias induced by conflict takes hold for the following trial. This is in contrast to recruitment of conflict counteraction which occurs within the same trial.

In order to look for neural correlates of history, we repeated our SE-SSM across subject TFCE based analysis with the parameter z_h. However, we found no statistically significant ($p < 0.05$, MST FWER corrected) activation. As an exploratory step, we also investigated whether the b_h parameter was correlated, and indeed found some regions of activation although not in ACC or DLPFC where they might have been expected[8]. We believe that the activation found in this analysis however, is likely due to the general correlation between b_h and b itself (Figure S5c) so we do not attempt to interpret this further.

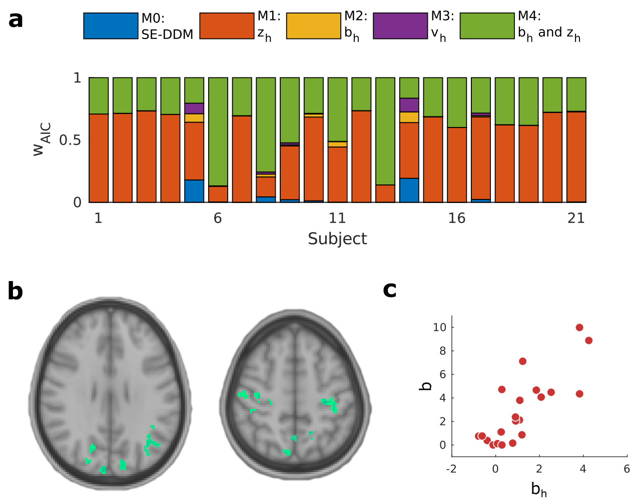

Figure S 5. History model, evaluation and results. (**a**) Model incorporating conflict history in the bias term has the highest w_{AIC}, improving over the SE-SSM, and being preferable to alternative history configurations. (**b**) While the z_h parameter does not seem to be related to brain activity when investigated with our across subject analysis, the parameter which is shown here in green b_h does. No negative activation was found with this analysis. (**c**) However this is likely due to the strong correlation between b_h and b (Spearman's CC = 0.76, $p = 1 \times 10^{-4}$).

References

1. McIntosh, J. R. & Mehring, C. Modifying response times in the Simon task with transcranial random noise stimulation. *Sci. Reports* **7**, DOI: 10.1038/s41598-017-15604-1 (2017).
2. Wiecki, T. V., Sofer, I. & Frank, M. J. HDDM: Hierarchical bayesian estimation of the drift-diffusion model in python. *Front. Neuroinformatics* **7**, DOI: 10.3389/fninf.2013.00014 (2013).
3. Navarro, D. J. & Fuss, I. G. Fast and accurate calculations for first-passage times in wiener diffusion models. *J. Math. Psychol.* **53**, 222–230, DOI: 10.1016/j.jmp.2009.02.003 (2009).
4. McIntosh, J. *Probing movement decision processes with transcranial current stimulation techniques*. Ph.D. thesis, Imperial College London (2016).
5. Brunton, B. W., Botvinick, M. M. & Brody, C. D. Rats and humans can optimally accumulate evidence for decision-making. *Science* **340**, 95–98, DOI: 10.1126/science.1233912 (2013).
6. Mumford, J. A., Turner, B. O., Ashby, F. G. & Poldrack, R. A. Deconvolving BOLD activation in event-related designs for multivoxel pattern classification analyses. *NeuroImage* **59**, 2636–2643, DOI: 10.1016/j.neuroimage.2011.08.076 (2012).
7. Gratton, G., Coles, M. G. H. & Donchin, E. Optimizing the use of information: Strategic control of activation of responses. *J. Exp. Psychol.* **121**, 480–506, DOI: 10.1037/0096-3445.121.4.480 (1992).
8. Kerns, J. G. Anterior cingulate and prefrontal cortex activity in an fMRI study of trial-to-trial adjustments on the Simon task. *NeuroImage* **33**, 399–405, DOI: 10.1016/j.neuroimage.2006.06.012 (2006).
